# Was It Mind-Reading?

**Published:** 1889-02

**Authors:** Amarala Martin

**Affiliations:** Cairo, Ill.


### WAS IT MIND READING?

BY AMARALA MARTIN.

Last October, while I sat with an independent slate writer, the following voluntary hint of the result of the presidential election was given: "Wait till after the *new* administration!" The question I asked had no reference whatever to politics, the election was of no interest to me, yet that message came on the slate and time has proven that there *is* to be a new administration.

The Sunday before the election a friend told me that he had received evidence through his own mediumship that the Republicans would gain the victory.

Here is another incident from my most intimate friend. I will give it in her own words : "You know, dear A, what tender love and devotion existed between myself and husband throughout all our acquaintance and how his death took out of my life all that had ever brightened it? Well, a year after my widowhood, with my heart yearning for even one token of remembrance from my lost one, I went one morning to see a trance medium. I had never met her before and there was no acquaintance or conversation between us prior to the seance. She at once described and named a number of my friends who had crossed the river, and gave me many truthful messages. She told me my own name and that of my husband also, giving me many consoling and hopeful words from him. But she startled me by declaring that he wished me to marry again as soon as a decent time had expired I was greatly shocked at this statement, but decided that the medium had come out of her trance and gone to fortune telling. So, bringing our seance to an abrupt close I went home and cried over my disappointment and humiliation. It seemed too cruel, I thought, that these tricksters should insult the memories of those we loved by giving such false messages.

The next morning I went to an independent slate writer, saying nothing of my former experience. Imagine then my surprise on receiving this message :

'My Dearest :—I am sorry that I made you so unhappy yesterday at Mrs. E's. I only sought your good. You are so lonely without me that I am unwilling to see you go through life alone. I want a companion who will appreciate you. I see that a man whom you know by reputation, (here followed a description of appearance and profession) will soon ask you to marry him. I wish you would do so. It would not interfere with our relations hereafter, as you are *mine* in spirit and ever will be. I dare not urge you more lest you doubt my constancy to you.

Your loving husband.'

In a few weeks after this message a gentleman answering the description given by my husband, asked me to be his wife. The request could not have come from a more unexpected source, as there were no preliminary compliments or courtships. But, dear A, I had and have but one love, and my soul looks out into eternity and calls its own."

What is the explanation of these several incidents? If mind reading, *whose* mind was read?

Cairo, Ill.

